# S100B Protein in the Nervous System and Cardiovascular Apparatus in Normal and Pathological Conditions

**DOI:** 10.1155/2010/929712

**Published:** 2010-11-10

**Authors:** Rosario Donato, Claus W. Heizmann

**Affiliations:** ^1^Department of Experimental Medicine and Biochemical Sciences, Section of Anatomy, University of Perugia, Via del Giochetto, 06122 Perugia, Italy; ^2^Department of Pediatrics, Division of Clinical Chemistry and Biochemistry, University of Zürich, Steinwiesstraße 75, 8032 Zürich, Switzerland

Accumulating evidence suggests that S100B, a Ca^2+^-binding protein of the EF-hand type, functions as a regulator of intracellular activities and as an extracellular signal. Within cells, S100B interacts with a relatively large number of target proteins thereby regulating their functions (Figure 1). While most of these interactions are Ca^2+^ dependent, in some instances S100B/target protein interactions are not. S100B is also secreted by certain cell types and released by activated/damaged/necrotic cells. Secreted/released S100B can affect cellular functions with varying effects depending on its local concentration.

S100B is not a ubiquitous protein, its expression being restricted to astrocytes, Schwann cells, ependymocytes, certain neuronal populations, adipocytes, chondrocytes, melanocytes, dendritic cells, muscle satellite cells, skeletal myofibers, arterial smooth muscle cells, and bronchial epithelium, in normal physiological conditions. However, the S100B cell expression pattern during prenatal and postnatal development might be different (there is limited information about this issue); S100B expression levels in certain cell types may be varied in response to extracellular factors; levels of S100B are high in several cancer cells; S100B expression may be induced in cardiomyocytes and arterial endothelium in response to norepinephrine. Serum levels of S100B in normal prepubescent and postpubescent human subjects are relatively high and low, respectively, and increases in S100B serum levels are found in physiological conditions (such as intense physical exercise) and in several pathological conditions (mostly, brain diseases, certain psychiatric disorders, melanoma, and heart infarction and insufficiency).

The present special issue of “*Cardiovascular Psychiatry and Neurology”* focuses on the brain-heart role of S100B. In the adult brain, S100B amounts to ~0.5% of cytoplasmic protein content and is most abundant in astrocytes (with an estimated concentration of ~10 *μ*M), where the protein is found diffusely in the cytoplasm and associated with microtubules, GFAP intermediate filaments, and intracellular membranes. Such a high concentration and its diffuse localization in the cytoplasm explain S100B's ability to interact with enzymes, enzyme substrates, scaffold/adaptor proteins, transcription factors, and cytoskeleton constituents thereby regulating energy metabolism, Ca^2+^ homeostasis, transcription, and cell shape, proliferation, differentiation, and motility (Figure 1). Besides, ~5% of S100B is being constitutively secreted by astrocytes; S100B secretion can be enhanced or reduced by a number of factors and/or conditions; in case of brain damage, large amounts of S100B are being passively released, with a fraction of the protein diffusing into the cerebrospinal fluid (CSF) and blood.

Whereas increases in the CFS and/or serum S100B content are taken as an indication of brain damage (yet increases in serum S100B content might point to nonbrain damage as well), a large body of evidence indicates that brain extracellular S100B behaves as a neurotrophin in normal physiological conditions and as a damage-associated molecular pattern (DAMP) factor upon massive release consequent to astrocyte activation or necrosis. In this latter case, S100B participates in the amplification of the brain inflammatory response by activating microglia and astrocytes and exerting toxic effects on neurons. RAGE (receptor for advanced glycation end products) has been identified as the receptor transducing both trophic and toxic effects of S100B in the brain and might act as a coreceptor supporting certain S100B effects on microglia (Figure 2).

As an intracellular regulator, S100B also participates in myocardium remodeling post infarction inhibiting the hypertrophic response in cardiomyocytes surviving the insult, and once released from necrotic cardiomyocytes, it acts as a DAMP factor causing cell death again via RAGE engagement. Adipocytes also release S100B in response to catecholamines with unidentified effects, though. Lastly, as a DAMP factor, S100B also participates in the pathophysiology of atherosclerosis stimulating vascular smooth cell proliferation and activating monocytes/macrophages.

The present issue of “*Cardiovascular Psychiatry and Neurology”* attempts to delineate the functional roles of S100B in the brain and the cardiovascular apparatus and to update the information about this multifaceted protein. We are confident that both cell biologists and clinicians will benefit by the reading of the papers presented therein.

*Rosario Donato* *Claus W. Heizmann*

## Figures and Tables

**Figure 1:**
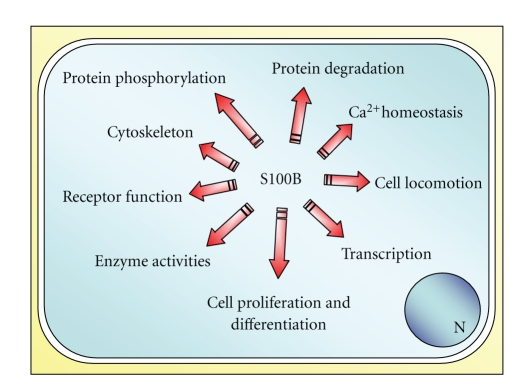
Schematic representation of intracellular regulatory effects of S100B.

**Figure 2:**
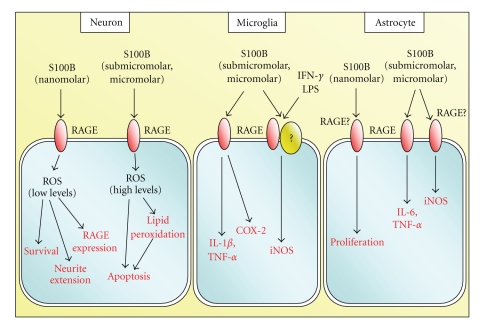
Schematic representation of extracellular regulatory effects of S100B on neurons, microglia, and astrocytes.

